# The transcriptional gradient in negative-strand RNA viruses suggests a common RNA transcription mechanism

**DOI:** 10.1371/journal.pcbi.1014441

**Published:** 2026-06-24

**Authors:** Connor R. King, Casey-Tyler Berezin, Brian Munsky, Jean Peccoud

**Affiliations:** 1 LSI Genomics, Princeton University, Princeton, New Jersey, United States of America; 2 School of Biomedical and Chemical Engineering, Colorado State University, Fort Collins, Colorado, United States of America; 3 Cell and Molecular Biology Program, Colorado State University, Fort Collins, Colorado, United States of America; University of York, UNITED KINGDOM OF GREAT BRITAIN AND NORTHERN IRELAND

## Abstract

Nonsegmented negative-strand RNA viruses (NNSV) are a diverse class of medically relevant viruses which display a conserved attenuation gradient in the transcription of their genomes. This gradient has been traditionally explained by the Stop-Start model which attributes attenuation to polymerase behavior at gene junctions. In this article, we evaluate an alternative explanation where the gradient arises from polymerase dynamics during transcription. We introduce the RNA Polymerase Association Mechanism (RAM) model, a coarse-grained stochastic framework that describes transcription using two parameters related to polymerase processivity and the ability of the polymerase to backtrack. The RAM model accurately reproduces transcriptional gradients across diverse NNSVs as well as in gene-shuffled VSV variants. Additionally, the inferred polymerase processivity appears correlated to the length of the viral genomes suggesting a conserved constraint on transcription across these viruses. While the RAM model does not account for all known molecular features of NNSV transcription, it provides a parsimonious and predictive framework for relating genome architecture and transcription. These results support the view that, in tandem with the traditional junction-centric mechanisms governing transcription, nonspecific attenuation mechanisms contribute to the NNSV transcriptional gradient and warrant closer inspection in future studies which could lead to better rational genome design in viral studies and biomedical applications.

## Author summary

Nonsegmented negative-strand RNA viruses (NNSVs), including Ebola virus, rabies virus, and vesicular stomatitis virus (VSV), share a common transcriptional gradient in which genes closer to the genome’s 3’ end are transcribed at higher levels than those farther away. The prevailing Start-Stop model attributes this gradient to polymerase behavior at gene junctions. The sequences located at these junctions control many essential aspects of transcription in these viruses such as initiation, termination, and post-transcriptional modifications (such as capping and polyadenylation). However, it is unclear whether the overarching transcriptional gradient in all NNSVs is the result of variation in the actions that occur at these junctions. Here, we propose a coarse-grained alternative framework that can recapitulate the observed gradient in these viruses without incorporating gene junction information. We propose the RNA-polymerase Association Mechanism (RAM) model, which describes transcription as a simple stochastic process where during transcription, the viral polymerase advances along the genome and either adds the next base to the end of the nascent transcript or dissociates from the nascent transcript resulting in premature termination of transcription. Our model requires only two parameters to reproduce transcriptional gradients across diverse NNSVs, including gene-shuffled VSV variants. We further show that the probability that the polymerase releases the transcript is correlated with genome length, suggesting a universal constraint on NNSV transcription. By shifting the focus from gene-junction sequences to polymerase dynamics, our work redefines how viral transcription is understood. These findings have broad implications for synthetic virology and vaccine design, providing a predictive framework for modifying viral gene expression, thereby enabling more precise engineering of viral vectors for therapeutic applications.

## Introduction

**Fig 1:**
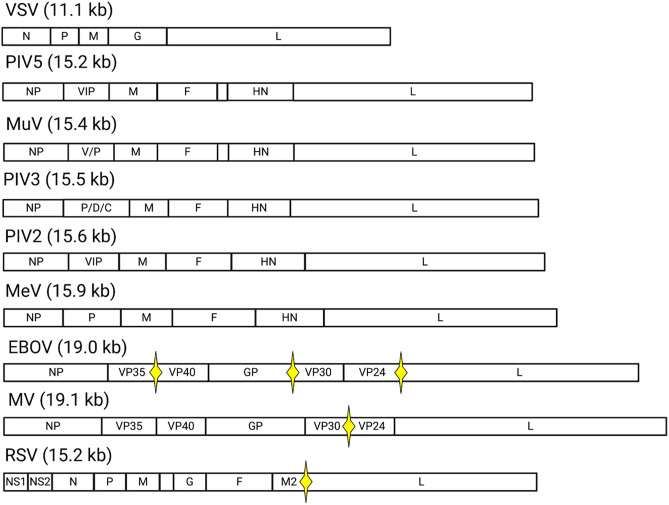
Genome Architecture of NNSVs. Graphical representation of the genomes of nonsegmented negative-strand RNA viruses (NNSVs) in 3’ to 5’ orientation with gene lengths to scale. Yellow stars indicate overlapping genes in the genome. The genes that lack labels are the regions containing the small hydrophobic (SH) gene. Common proteins present in most of these species are the N/NP = nucleocapsid, P = phosphoprotein, M = matrix protein, G/GP = glycoprotein, L = RNA-dependent RNA polymerase, F = fusion protein, and HN = hemagglutinin-neuraminidase protein.

Nonsegmented negative-strand RNA viruses (NNSVs), including Ebola (EBOV), rabies virus, and vesicular stomatitis virus (VSV), are a diverse class of viruses that are commonly used in biomedical applications such as vaccine vectors [1]. These viruses possess a single RNA strand encoding tightly packed genes (Fig 1) and their transcriptional output is linked to gene order. In the 1970s, UV inactivation studies in VSV suggested a single polymerase initiates at the 3′ end and transcribes each gene sequentially [2–4]. Although early data showed similar protein levels [5], differences in protein size implied non-equimolar expression. This difference in relative expression was characterized at both the protein and transcript level to reveal a 3’ to 5’ gradient in expression, suggesting that the control was at the transcription level [6]. This gradient led researchers to suggest two hypotheses.

Iverson and Rose rationalized that since the transcripts of VSV had been shown to have roughly equal degradation rates [7], the difference in relative expression was due to actions in the genome either at specific locations (gene junctions) or nonspecifically [8]. They designed probes to quantify the relative amounts of the 3’ and 5’ end of transcripts as they hypothesized that nonspecific attenuation would result in a gradient at the transcript level while specific attenuation would show equimolar levels of these fragments. This paper also established that at most gene junctions there is a 29–33% drop while the junction before the L polymerase causes a 80–90% decrease in transcription. This hypothesis was soon dubbed the “Stop-Start Hypothesis” [9] and was extended to many other NNSVs, including EBOV [10,11], Marburg virus (MV) [10,12], parainfluenza virus type 2 (PIV2), parainfluenza virus type 3 (PIV3) [13,14], parainfluenza virus type 5 (PIV5) [13,15], mumps virus (MuV) [13,16], measles virus (MeV) [17], and respiratory syncytial virus (RSV) [18,19]. While this was a hot topic for many years, the debate over this phenomenon has quieted and to this day, the Stop-Start Hypothesis remains the dominant hypothesis [20].

Much of the literature has focused on dissecting what sequences at these gene junctions control the level of attenuation observed. Particular focus has been interested in the gene junction prior to the L gene as in general, we tend to see consistent levels of attenuation for all junctions at around 30% except for the L gene which exhibits about 80–90% attenuation. The cause of this has remained elusive, as in some viruses this attenuation can be strongly attributed to gene junctions while others it cannot. Indeed, in rabies virus we can see that the level of attenuation is strongly tied to the intergenic sequence located between the stop and start signal sequences and this has been used to generate variants of rabies virus that overexpress the L polymerase [21]. Additionally, RSV also has been shown to have termination depend strongly on the gene junction present, but intriguingly there is no effect from the very structurally diverse intergenic regions [18,22].

On the other hand, some viruses like EBOV do not offer such a clean explanation. For EBOV, attenuation at the L junction is unremarkable, while we do see an effect on the NP/VP35 and VP30/VP24 junctions [23]. Although, it should be noted that the inclusion of the 5’ and 3’ UTRs might have influenced these experiments as they may impact the stability of transcripts. Another intriguing study using PIV5 looked at attenuation from different gene junctions in the context of expressing HN and L. Three different levels of attenuation were observed across the 6 genomes with the M-F, F-SH, and SH-HN junctions producing higher attenuation than the HN-L junction. Even more perplexing, these variations in attenuation rates were not observed when the genes in the minigenome were swapped for F and V/P [15].

These works suggest that there are several mechanisms that come together to orchestrate the gradient observed in literature. Indeed, some of these mechanisms that have come into focus have more to do with intrinsic polymerase properties and could be viewed as a source of nonspecific attenuation. The efficiency of 5’ capping has gained more focus in recent years where it has been shown that the formation of the 5’ cap during transcription is necessary for the proper elongation and termination of transcription [17,24–26]. When this process fails, it results in the production of truncated transcripts. Indeed such cap-less truncated transcripts had even been noted in RSV a decade earlier [27]. Something intriguing here is the observation of truncated transcripts, the absence of which was central to the initial formation of the stop-start model.

**Fig 2:**
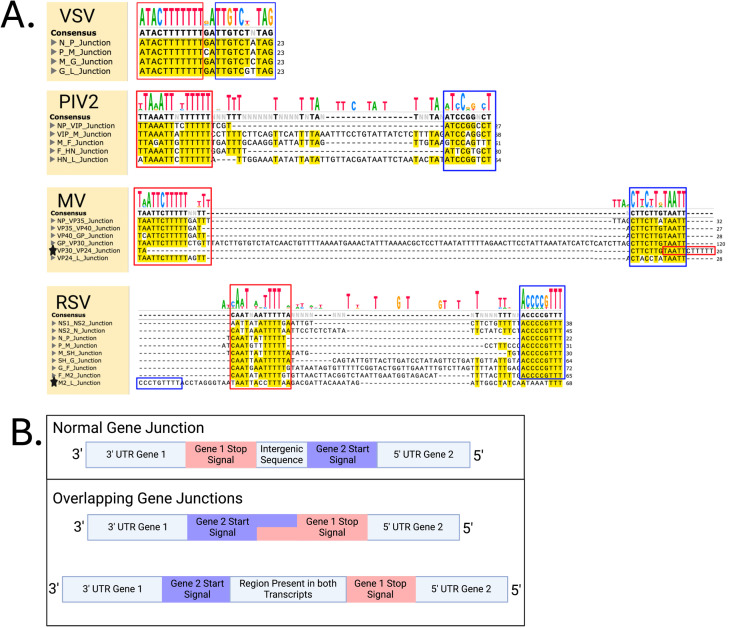
Gene Junction Diversity of NNSVs. **A.** Alignments of gene junctions for 4 NNSVs. Each junction has blue and red boxes highlighting the conserved transcriptional start and stop sequences respectively. Black stars to the left of the sequence indicate that the junction is an overlapping junction and so the locations of the start and stop sequences are inverted compared to the others. **B.** Cartoon diagram of the types of gene junctions observed in NNSVs and overlapping gene junctions. The UTRs in this figure refer to the untranslated regions of the transcripts that are produced.

As such, there may be value in revisiting the study of NNSV transcription from the lens of nonspecific attenuation. Indeed, it has been shown that in measles virus the slope of the transcriptional gradient can be altered based on the affinity between the X domain of the P protein (which is part of the transcription complex) and the C terminal tail of the Nucleoprotein [28]. Revisiting these proposed mechanisms is also warranted as the template dissociation-based mechanism appears to be at odds with the structure of the polymerase-template complex. The L polymerase completely encircles the genome while moving down the genome [29]. So, the polymerase would need to undergo a dramatic rearrangement that is highly unlikely in order to dissociate from the template. Moreover, junction sequences are highly diverse, even within the same virus (Figs 2A, S1 Fig.). The initiation sequence itself is poorly conserved, with the minimal required motif being 3’-UYGnnnnnnn-5’ [30]. This raises the question of how this consistent gradient that is present across all NNSVs can be the direct result of such a diverse array of sequences.

In this article, we develop a model of transcription that attributes the amount of attenuation at each junction due to the distance the polymerase must travel from the 3’ end of the genome to the gene’s termination signal. First, we demonstrate that the mechanism is consistent with a wide variety of NNSV transcriptional gradient datasets. Then we show that the resulting predictions from a model fit to VSV transcriptional data is as consistent with the observed protein expression gradients in gene shuffled VSV variants as the Stop-Start model despite having fewer parameters (1 instead of 4). Additionally, there is even some weak evidence that the RAM model produces slightly better predictions than the Stop-Start model when considering the expression of genes in gene-shuffled VSV variants. Given that it is unlikely that the polymerase dissociates from the genome template [29], it is unclear why moving down the genome results in fewer transcripts being produced. It is possible that “losing” the nascent transcript results in a polymerase state that is unable to continue transcription until it restarts all over again at the 3’ entry site. Indeed, there are extensive regulatory interactions that have been characterized between the nascent RNA and the RNA polymerase [31]. Since we attribute the amount of attenuation at each junction to the polymerase’s ability to remain associated with the nascent transcript during transcription, we name this model the RNA Polymerase Association Mechanism (RAM) model.

## Results

### Modeling NNSV transcription as a walk

**Fig 3:**
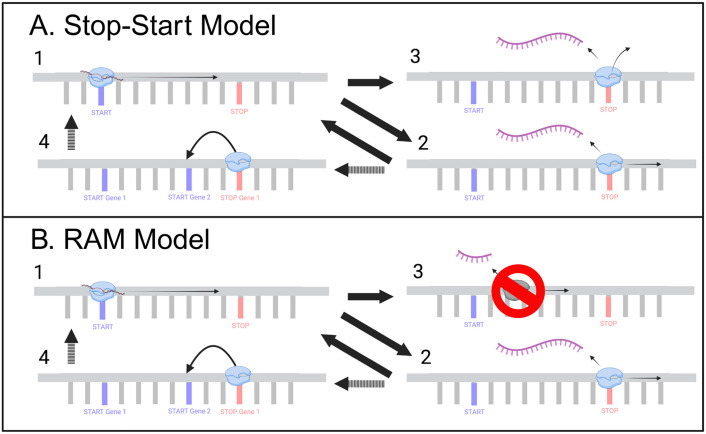
Diagram of Stop-Start and RAM Models. (**A.**) Stop-Start Model and (**B.**) RAM Model. In both models transcription begins at the 3’ most end of the genome (state 1) where the polymerase recognizes the start signal and initiates transcription. The polymerase then proceeds down the genome to the termination signal (state 2) and releases the synthesized transcript. For standard gene structures, the polymerase reinitiates at the next downstream start signal (state 1). If there is an overlap, the polymerase must move backwards to state 4 before proceeding to state 1. The models differ in where the attenuation we observe comes from. In the RAM model, the polymerase slips and releases the nascent transcript, entering a state that can no longer recognize transcription start signals (state 3). In the Stop-Start model, the polymerase dissociates from the genome at the junction (state 3). Although this too may instead be due to some sort of conformational change into an inactive polymerase conformation at these junctions. These two models are not necessarily mutually exclusive. Both processes are likely to be occurring. Created in BioRender. King, **C.** (2026) https://BioRender.com/z9eob9v.


The RAM model consists of a single polymerase performing a walk beginning at the 3’ end of the genome (Fig 3). In each step, the polymerase takes one of two actions. The polymerase either takes a single base step forward and continues productive transcription or it dissociates from the transcript and enters a nonproductive state where it can no longer initiate transcription. The probabilities associated with these actions will be referred to as p_maintain_ and p_drop_, respectively. Since p_drop_ is the complement of p_maintain_, these represent a single parameter in this model. The relative level of expression of most genes (p_express_) can be calculated from the distance of the stop signal for the gene from the 3’ end of the genome (n) using the equation:

$$\begin{array}{c} {\text{p}}_{\text{express}}\;=\;{{(\text{p}}_{\text{maintain}})}^{\text{n}} \end{array}$$

Where n is the number of bases that the termination sequence is from the 3’ end of the genome. It is important to note that some NNSVs have overlapping genes, where the transcriptional start signal of one gene is located within the upstream gene (Fig 2). In cases where overlapping occurs and the transcription start site of a gene (Gene 2) is located within an upstream gene (Gene 1), then the transcription of Gene 2 only occurs if the polymerase backtracks back to the start signal of Gene 2 after finishing transcription of Gene 1 [20,32]. We characterize this using one additional parameter called p_backtrack_. In these cases p_express_ is calculated using this formula:


$$\begin{array}{c} {\text{p}}_{\text{express}}\;=\;{{(\text{p}}_{\text{maintain}})}^{\text{n}}\;\times \;{\text{p}}_{\text{backtrack}} \end{array}$$

The parameters p_maintain/_p_drop_ and p_backtrack_ were determined by the fitting the model to maximize the likelihood of the data given the model under the assumption that their values were between 0 and 1, and posterior uncertainties in parameters were quantified using Markov Chain Monte Carlo (MCMC) algorithm (S2 Table) [33,34]. The data used in this article are very diverse, including sequencing data from Illumina sequencing and direct RNA sequencing as well as blotting-based methodologies. The code for fitting the models can be found in Notebook1 on the GitHub associated with this article.

### The RAM model fits the transcriptional gradient of diverse NNSVs

**Fig 4:**
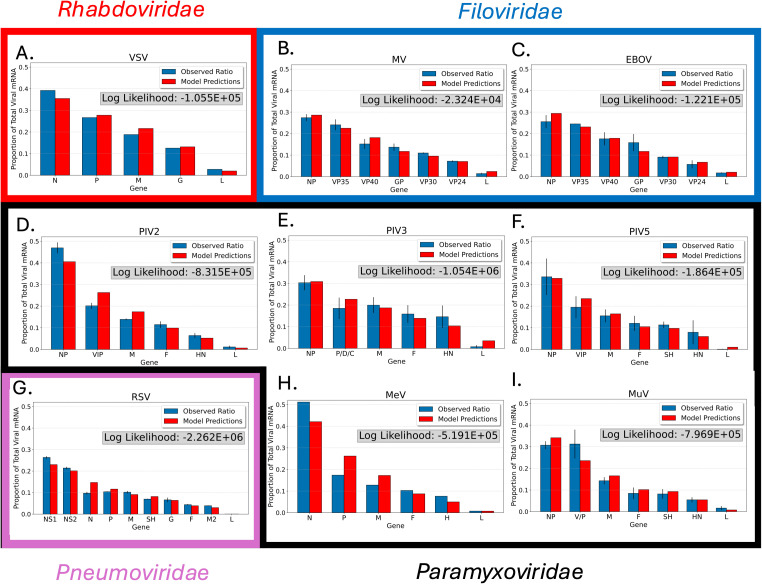
The RAM Model Accurately Predicts Transcriptional Gradients Across NNSV Species. Model predictions of the transcriptional gradient of NNSVs using the RAM model (red bars) compared to published data (blue bars). These predictions are across predominant families of NNSVs: *Rhabdoviridae* **(A)**, *Filoviridae* **(B and C)**, and *Paramyxoviridae* (**D-F, H, I**), and *Pneumoviridae* **(G)**. Black error bars are 4 standard deviations in length, or were excluded if standard deviations were not available for the data. The log likelihood is included for every model.

The RAM model was fit to the observed gradients from 9 NNSVs of the *Pneumoviridae*, *Paramyxoviridae*, *Filoviridae*, and *Rhabdoviridae* families (Fig 4, S2 Table). For *Rhabdoviridae*, the model was fit to the original dataset used to describe the ratio of VSV expression in 1981 [8]. For *Filoviridae*, EBOV and MV were used [35]. For *Pneumoviridae*, RSV was used [36]. For *Paramyxoviridae*, data from MeV, MuV, PIV2, PIV3, and PIV5 were used [13,37]. It is important to note that the published data associated with the transcriptional behavior of RSV are inconsistent. Some articles propose an entirely different gradient behavior of RSV with several inconsistencies when compared to the characteristic simple gradient [32,38]. In this article, the RSV gradient that most closely resembled the characteristic NNSV gradient was selected, as this is the one that is most consistent with the current understanding of NNSV transcription in literature.

The RAM model was able to achieve high-quality fits for all these viruses (Fig 4). The confidence in parameter p_maintain_ associated with each virus are well-determined (S2 Table). In general, the p_backtrack_ parameter is well-determined in the viruses that have overlaps in their genomes while it appears underdetermined in those that do not (S2 Table). This is not surprising as models of viruses without overlaps never actually utilize this parameter. As such, in these scenarios, this model can be reduced to a single parameter. In viruses that have overlapping genes, in order for the downstream gene of an overlapping junction to be transcribed the polymerase must backtrack to the transcriptional start signal upon completion of transcribing the upstream gene. This should lead to additional attenuation that comes from the parameter p_backtrack_ in these viruses. However, the junctions in each virus appears to behave substantially different from one another with RSV demonstrating the largest amount of additional attenuation (94.15-94.33% more) attributed to the overlap while MV and EBOV had very little if any attenuation (1.6-20.05% more and 0-.065% more respectively. In RSV, it has been shown that part of this additional attenuation is due to premature stoppage of transcription at the internal stop signal [39]. It is possible that since the overlap in MV and EBOV are less dramatic than in RSV (Figs 2A, S1 Fig.) this effect does not occur in these viruses. Alternatively, it could be due to the larger distance the polymerase must travel.

### Genome size is correlated to polymerase processivity

**Fig 5:**
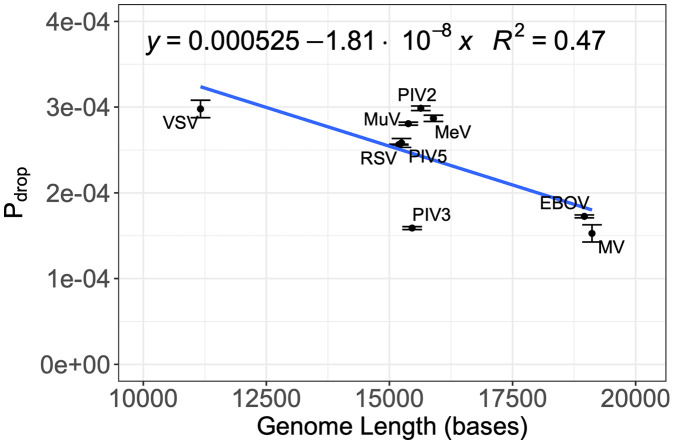
The Processivity of Viral Polymerases is Correlated to Genome Length. This figure shows a linear model showing how the probability of the polymerase releasing the nascent transcript at each step (P_drop_) decreases as the length of the genome increases. The estimated slope (-1.81 x 10^-8^ ± 1.70 x 10^-8^) and intercept (5.25 x 10^-4^ ± 2.72 x 10^-4^) are both statistically significant (t(7) = -2.50, p = .041 and t(7) = 4.58, p = .003 respectively). The dots and error bars are the estimates and 95% confidence intervals respectively for the value of p_drop_ for each virus.

Since p_drop_ is the probability of the polymerase releasing the nascent transcript at any point, it can be viewed as a type of polymerase processivity. Intriguingly, there appears to be a negative correlation between the value of p_drop_ and the length of the genome (Fig 5). This correlation suggests that for every base increase in the genome, the probability of the polymerase releasing the transcript at every step down the genome decreases by about 1.79 x 10^-8^ ± 1.72 x 10^-8^ (p-value = .044, t-test statistic = 2.45). This observation is reasonable as all polymerases must enter the 3’ end of the genome and so the polymerase must stay active for longer stretches in order for genes at the 5’ end of longer viral genomes to be expressed.

### The RAM model accurately predicts gene expression in reorganized genomes

Historically, VSV has been studied using “gene-shuffled” variants where the position of genes is shifted to leverage the transcriptional gradient to predictably alter transcription rates [40,41]. Here, the RAM model and the Stop-Start model were both evaluated on their ability to predict how shuffling the position of VSV’s genes alters the observed expression ratio of the genes [41,42]. The Stop-Start model used here is model with 4 parameters, one for each junction. The amount of attenuation expected at each junction is essentially the amount of attenuation observed in the training dataset. The available data is all at the protein level, however, it is generally understood that the gradient in the expression of VSV genes at the transcript level also occurs at the protein level, with some potential to deviate due to other mechanisms that may be controlling protein expression [40–42].

**Fig 6:**
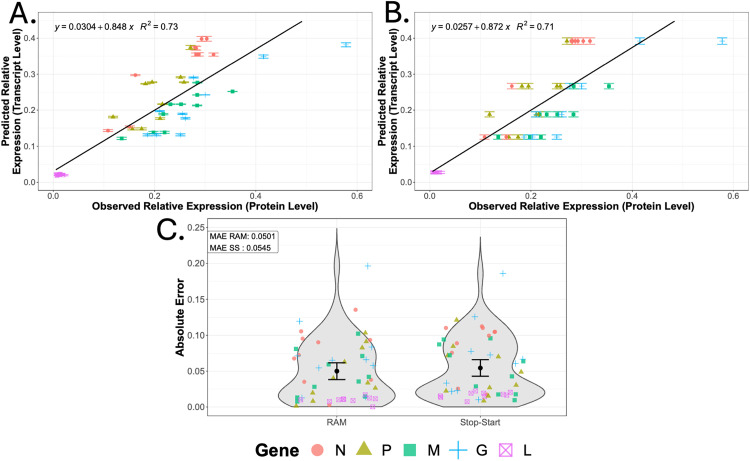
The RAM Model Accurately Predicts Gradients Across Gene-Shuffled VSV Variants. This figure shows the linear fits for the predicted relative mRNA transcript levels in different VSV gene-shuffled variants for the (**A.)** RAM Model and (**B.**) Stop-Start Model (y-axis) compared to the observed protein expression levels of experimentally constructed gene-shuffled VSV variants from 2 previous publications, Ball et al. 10.1128/jvi.73.6.4705-4712.1999 and Flanagan et al. 10.1128/jvi.74.17.7895-7902.2000 (x-axis). The slopes of both the RAM model (0.848 ± 0.152) and the Stop-Start model (0.872 ± 0.161) are statistically significant (t(48) = 11.25, p = 4.67 x 10^-15^ and t(48) = 10.89, p = 1.46 x 10^-14^). **C.** Shows the distribution of absolute errors between predicted and observed values between both models along with the Mean Absolute Error. The differences between these errors is not statistically significant (t(49) = -1.74, p = 0.087).

Both models were first fit to the Iverson and Rose VSV transcriptional gradient data [8] using MCMC. Then, to evaluate the models parameters were drawn from the posterior distributions. Each of these draws was used to simulate the models and the average of these predictions was used to calculate the Mean Absolute Error between the predictions and observed expression levels for gene-shuffled variants from two datasets [41,42]. Both models do well predicting the relative expression levels (Fig 6A and B) and show a statistically significant correlation between the predicted expression levels. This is consistent with the previous statement that the expression gradient is carried on to the protein level and may occur at a nearly 1:1 level. However, there do appear to be some deviations between predicted and observed expression levels. It is possible that this is due to differing environmental factors, such as the degradation rate [43,44].

When looking at the error in these two models, while it is not statistically significant there is a very weak benefit from using the RAM model which has an MAE of 0.0501 as opposed to the Stop-Start model which has an MAE of 0.0545 (Fig 6C). It is likely that this is since the Stop-Start model is much more susceptible to overfitting since the number of variables is equal to the number of gene junctions (4) while the RAM model relies on only 1 parameter.

## Discussion

The model proposed here provides a simplified, quantitative approximation of the transcriptional gradient in NNSVs without explicitly incorporating junction-specific regulation. The RAM model can recapitulate the transcriptional gradient in a diverse array of viruses using only one (and at most two) parameters (Fig 4) providing a more parsimonious model as opposed to the Stop-Start model which has one parameter for each gene junction. This simplicity lends the model to be less susceptible to overfitting, the effects of which can be seen in the minor advantage the model has over the Stop-Start model when predicting the relative expression of genes in gene-shuffled VSV variants (Fig 6C).

The RAM model’s robustness across diverse datasets ranging from traditional methods that use ^3^H-uridine to quantify viral RNAs [8] to next-generation sequencing data [35] further supports its broad applicability. The model presented in this article is effectively a successor to the non-specific attenuation model that had been previously discarded in the early 1980s [8]. By showing that a simple polymerase-based stochastic process can reproduce the transcriptional gradient across multiple NNSVs, our results suggest that these observed polymerase dynamics may represent a conserved underlying constraint in viral transcription. A result supported by the correlation between polymerase processivity and genome length (Fig 5).

Despite the benefits of the RAM model, there are limitations. The RAM model inherently does not capture all aspects of NNSV transcription. There are many cases where the gene expression gradient is strongly impacted by the gene junctions, such as rabies virus [21] and RSV [18,22]. These observations are more consistent with the Stop-Start model and highlight why the Stop-Start model is still a central model in NNSV research. Rather than replacing the Stop-Start model, the RAM model represents a complementary explanation that captures baseline attenuation and encourages the investigation of nonspecific mechanisms also known to be at work, such as premature termination due to failed 5’ capping [17,24–26].

However, open questions remain. One of the major implications of the RAM model is the generation of truncated transcripts. Such transcripts have a complicated history in the NNSV transcriptions space. On one hand, Iverson and Rose used the lack of observed truncated transcripts as evidence for refuting a nonspecific attenuation mechanism [8]. On the other, truncated transcript have been observed in RSV [27] and when studying the role of 5’ capping in [17,24–26]. It is unclear if these discrepancies are due to biological differences in the viruses or methodological differences in these studies. It also should be noted that in most studies the transcript levels observed are the steady-state levels. So, in some cases the absence of truncated transcripts could be due to reduced stability from a lack of 5’ caps and/or polyadenylated tails [13,45]. Another alternative interpretation is that this is an artifact of the assumption that there is a uniform probability of attenuation across the genome. Rather, the dissociation of the polymerase from the transcript may be limited to specific stages in transcription. For example, it may be that the phenomenon associated with this is in fact the aforementioned 5’ capping-dependent transcriptional termination mechanism. However, why the amount of attenuation would be correlated with the length of the gene is unclear.

To resolve these discrepancies, we advocate moving beyond minigenome assays toward large-scale variant libraries. While this was extremely challenging during these initial studies, it is now possible to leverage homology-based methods to generate large panels of variants [46,47]. Gene-shuffled and junction-swapped constructs, analyzed by qPCR or RNA-seq (in the absence of poly-A pulldowns), could reveal the individual contributions of gene order, junction sequences, and polymerase behavior. Such data would allow for a more rigorous understanding of NNSV transcription and help discern what mechanisms are at play. However, we should not neglect the fact that it is highly likely that both the specific and nonspecific mechanisms discussed in this article participate together in the regulation of transcription and therefore the emergence of a transcriptional gradient in NNSVs.

Overall, this work brings attention back to the potential for a per-base attenuation mechanism that may also be at work in the NNSV transcriptional gradient. At minimum, this model provides a useful null control for the assumed gradient for any NNSV with an uncharacterized gradient. At best, this brings back the idea that there should be some revisitation to a per-base nonspecific attenuation mechanism that was discarded very early on in the study of NNSV transcription. Such per-base attenuation could be exceedingly useful in the biomedical community as it may allow for improved calibration of viral transcriptional profiles in therapies. For example, it has been a major point of focus to understand how the alteration of transcription rates influences the fitness of the VSV, primarily to attenuate it for medical applications [41,44,48]. Our model suggests new design strategies beyond gene shuffling (namely, gene overlaps and gene length modulation). Considering overlapping genes as binary variables yields 1,920 possible VSV variants. Incorporating gene-length changes, which might influence transcription rates and are feasible due to VSV’s tolerance for large genome insertions [49–51], dramatically expands the design space. Together, gene order, overlap, and length could provide unprecedented control over viral transcription. This would enable not just understanding of viral biology but also the rational design of attenuated or customized NNSV variants with finely tuned expression profiles.

## Materials and methods

### Identification of gene junctions

Gene junctions were identified in reference genomes by using literature reported gene junctions as a guide (S1 Table) [15,22,23,37,52–56]. Junctions were then aligned in SnapGene using MUSCLE. Reference genomes, junctions, and alignments can be found in Folder4 on GitHub.

### Parameter estimation

To estimate parameters, the log likelihood for observing the data based on the model-predicted ratios was maximized. The minimize function from SciPy was utilized. Each model was fit 10,000 times starting at a value between 0 and 1. The parameters chosen for downstream analyses were the ones associated with the largest log likelihood. Confidence in parameters was assessed using MCMC algorithm [33,34]. The code for fitting models and performing MCMC can be found in Notebook1 on GitHub. The code for then making predictions for gene shuffled variants can be found in Notebook2.

### Linear regression

Linear regression was performed in R using the base lm function. The performance package was used to test model assumptions of normality, homogeneity of variance, and linearity. No violations were observed. The code for fitting the linear regression model connecting polymerase processivity and genome length can be found in the Markdown1 file on GitHub. The code for fitting the linear regression model connecting RAM model predicted expression and observed expression for gene shuffled variants can be found in the Markdown2 file on GitHub.

## Supporting information

S1 Fig Alignments of Gene Junctions for 9 NNSVs. Each junction has blue and red boxes highlighting the conserved transcriptional start and stop sequences respectively. Black stars to the left of the sequence indicate that the junction is an overlapping junction and so the locations of the start and stop sequences are inverted compared to the others.
(TIFF)

S1 Table Literature Reported Gene Junction Consensus Sequences. (PDF)

S2 Table Best Fit Parameter Along with Confidence Statistics from MCMC for RAM Model. (PDF)

S3 Table GenBank Accession Numbers for Nonsegmented Negative-Stranded RNA Virus Genomes. (PDF)
