# Supplementary figures and images for: The transcriptional gradient in negative-strand RNA viruses suggests a common RNA transcription mechanism
Source: PLoS Comput Biol. 2026 Jun 24;22(6):e1014441. doi: 10.1371/journal.pcbi.1014441 (PMC13313335; doi:10.1371/journal.pcbi.1014441)

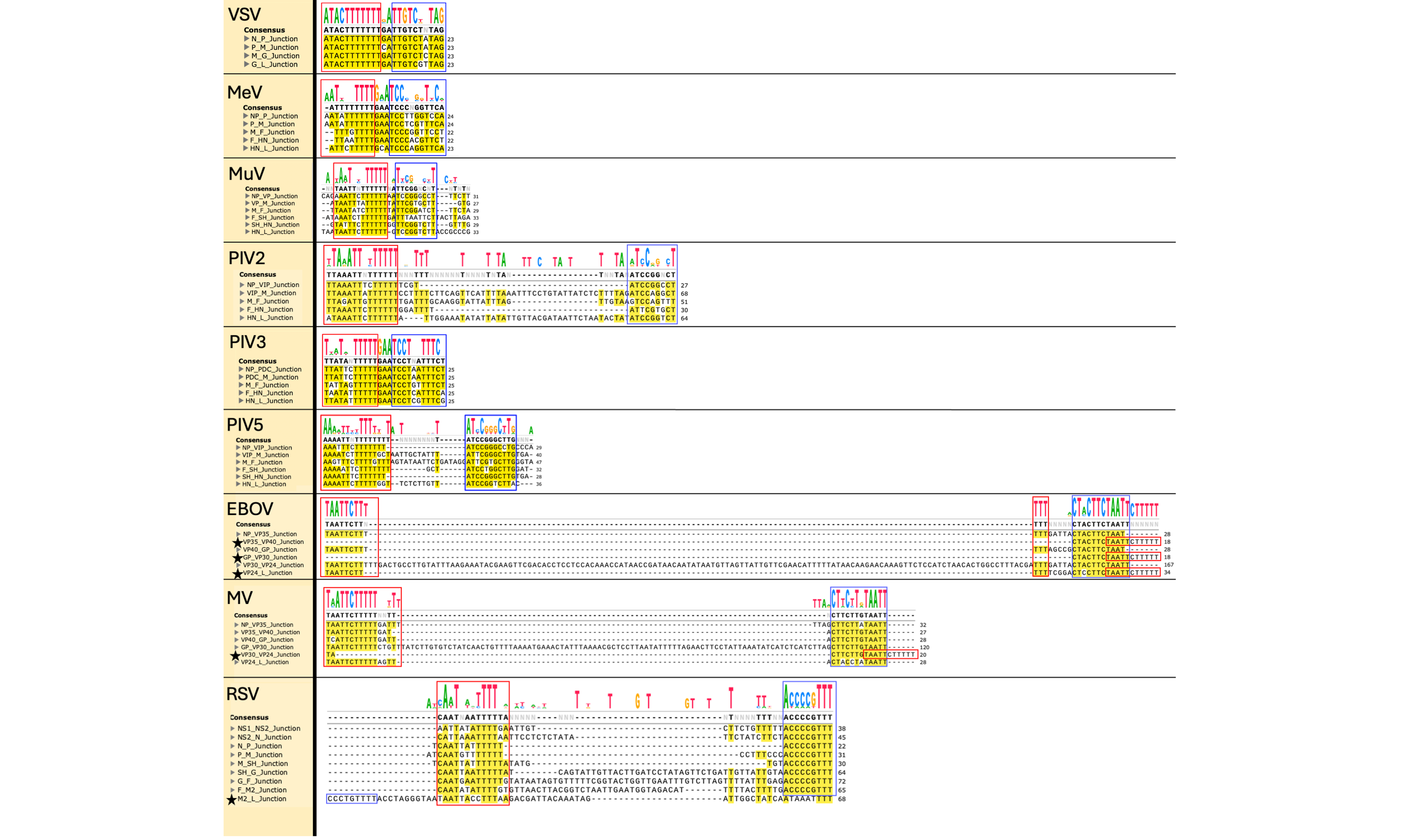

Supplement: S1 Fig — Each junction has blue and red boxes highlighting the conserved transcriptional start and stop sequences respectively. Black stars to the left of the sequence indicate that the junction is an overlapping junction and so the locations of the start and stop sequences are inverted compared to the others. (TIFF) [file pcbi.1014441.s001.tiff]
