# Supplementary material for: The transcriptional gradient in negative-strand RNA viruses suggests a common RNA transcription mechanism
Source: PLoS Comput Biol. 2026 Jun 24;22(6):e1014441. doi: 10.1371/journal.pcbi.1014441 (PMC13313335; doi:10.1371/journal.pcbi.1014441)
Supplement: S1 Table — (PDF) [file pcbi.1014441.s002.pdf]

Table S1. Literature Reported Gene Junction Consensus Sequences

Virus	Start Sequence	Intergenic Sequence	Stop Sequence	Reference DOI	Citation Number
Vesicular Stomatitis Virus	3'-UUGUCNNUAG-5'	3'-GA-5'	3'-AUACUUUUUUU-5'	10.1016/0092-8674(80)90515-2	54
Measles Virus	3'-UCCNNNNUNCN-5'	3'-GAA-5'	3'-NNUNU$_{4-6}$-5'	10.1002/j.1460-2075.1987.tb04808.x	38
Mumps Virus	3'-UCUCUGNNUCNU-5'	Not Conserved	3'-AUANUUACU$_{6-7}$-5'	10.1099/0022-1317-69-11-2893	55
Parainfluenza Virus 2	3'- UCCGGNCU-5'	Not Conserved	3'-UUAAAUUCU$_{5-7}$-5'	10.1093/nar/19.10.2739	56
Parainfluenza Virus 3	3'-UCCUNNUUUCU-5'	3'-GAA-5'	3'-UUNAUNNUUUUUU-5'	10.1128/JVI.59.3.646-654.1986	57
Parainfluenza Virus 5	3'-A$_{2-4}$U$_{1-3}$CU$_{4-7}$-5'	Not Conserved	3'-UNCGGGCUUGN -5'	10.1128/jvi.73.8.6228-6234.1999	15
Ebola Virus	3'-CUACUUCUAAU-5'	Not Conserved	3'-UAAUUCU$_{5-6}$-5'	10.1016/j.virusres.2009.02.005	24
Marburg Virus	3'-NNCUNCNUNUAAUU-5'	Not Conserved	3'-UAAUCUUUUUU-5'	10.1016/0168-1702(92)90027-7	58
Respiratory Syncytial Virus	3'-CCCCGUUUA-5'	Not Conserved	3'-UCAAUUAUUUUUU-5'	10.1128/jvi.70.9.6143-6150.1996	23
