# Supplementary material for: The transcriptional gradient in negative-strand RNA viruses suggests a common RNA transcription mechanism
Source: PLoS Comput Biol. 2026 Jun 24;22(6):e1014441. doi: 10.1371/journal.pcbi.1014441 (PMC13313335; doi:10.1371/journal.pcbi.1014441)
Supplement: S2 Table — (PDF) [file pcbi.1014441.s003.pdf]

Table S2. Best Fit Parameter Along with Confidence Statistics from MCMC for RAM Model

Virus	Parameter	Best Fit Estimate	Best Fit Log Likelihood	2.5% Quantile	97.5% Quantile
VSV	$p_{maintain}$	0.99970	-1.06E+05	0.99969	0.99971
VSV	$p_{backtrack}$	0.37232	-1.06E+05	0.00297	0.79360
MeV	$p_{maintain}$	0.99971	-5.19E+05	0.99971	0.99972
MeV	$p_{backtrack}$	0.25626	-5.19E+05	0.01834	0.53623
PIV2	$p_{maintain}$	0.99970	-8.32E+05	0.99970	0.99970
PIV2	$p_{backtrack}$	0.59228	-8.32E+05	0.40021	0.83575
PIV3	$p_{maintain}$	0.99984	-1.05E+06	0.99984	0.99984
PIV3	$p_{backtrack}$	0.87515	-1.05E+06	0.58381	0.96385
PIV5	$p_{maintain}$	0.99974	-1.86E+05	0.99974	0.99975
PIV5	$p_{backtrack}$	0.58308	-1.86E+05	0.15806	0.93039
MuV	$p_{maintain}$	0.99972	-7.97E+05	0.99972	0.99972
MuV	$p_{backtrack}$	0.17845	-7.97E+05	0.05535	0.28851
EBOV	$p_{maintain}$	0.99983	-1.22E+05	0.99983	0.99983
EBOV	$p_{backtrack}$	0.99945	-1.22E+05	0.99935	1
MV	$p_{maintain}$	0.99985	-2.32E+04	0.99984	0.99986
MV	$p_{backtrack}$	0.90312	-2.32E+04	0.79949	0.98403
RSV	$p_{maintain}$	0.99974	-2.26E+06	0.99974	0.99974
RSV	$p_{backtrack}$	0.05741	-2.26E+06	0.05676	0.05855
