# Supplementary material for: The transcriptional gradient in negative-strand RNA viruses suggests a common RNA transcription mechanism
Source: PLoS Comput Biol. 2026 Jun 24;22(6):e1014441. doi: 10.1371/journal.pcbi.1014441 (PMC13313335; doi:10.1371/journal.pcbi.1014441)
Supplement: S3 Table — (PDF) [file pcbi.1014441.s004.pdf]

Table S3. GenBank Accession Numbers for Nonsegmented Negative-Stranded RNA Virus Genomes.

Virus	GenBank Accession
Vesicular Stomatitis Virus	J02428
Measles Virus	NC_001498
Mumps Virus	JN012242
Parainfluenza Virus 2	KM190939
Parainfluenza Virus 3	NC_075446
Parainfluenza Virus 5	NC_006430
Ebola Virus	NC_002549
Marburgvirus	DQ447653
Respiratory Syncytial Virus	M74568
