# Supplementary material for: The transcriptional gradient in negative-strand RNA viruses suggests a common RNA transcription mechanism
Source: PLoS Comput Biol. 2026 Jun 24;22(6):e1014441. doi: 10.1371/journal.pcbi.1014441 (PMC13313335; doi:10.1371/journal.pcbi.1014441)
Supplement: Attachment — Submitted filename: PLOS_Responses_v1.pdf [file pcbi.1014441.s005.pdf]

Reviewer #1:

Comment 1: Figure 1 needs more explanation and references in the main text (Fig. 1A and Fig. 1B instead of Fig. 1). Especially, Fig. 1B is unclear. What are the consequences of those different structures?

Response: In the revised manuscript, Figure 1 has been split and expanded for clarity (lines 60-69). The junction structures are now presented in Figure 2, which includes annotated sequence alignments (panel A) and schematic representations of overlapping and standard gene junctions (panel B) (lines 100-107).

We now discuss the biological implications of these differences in the Introduction (lines 92-95), highlighting how overlapping genes challenge the traditional Start-Stop model.

Comment 2: The method section needs more explanation and details. Since this is the data you are fitting your model to, it is unclear how it looks, what was measured (what does “gradient” precisely mean?), and where the data comes from.
What is the model? You describe the model in the results section, but the equations and parameter ranges you used for model fitting are missing. Explain the different methods in at least one sentence so the reader understands what you were doing and why you were doing it.

Response: We have expanded the Data section in Materials and Methods to clarify the origin of datasets (lines 297-305). These include RNA-seq and blotting-based methods across 9 NNSVs. The term "gradient" is now clearly defined as the normalized relative expression of mRNAs ordered from 3' to 5' along the genome.

Comment 3: The equations are missing in the results section where you describe the model (lines 114 and 121). What are parameter value assumptions?

Response: In the initial submission, the equations were unintentionally omitted. They are now present in the Results section (lines 136-146), including the mathematical form of the expression probabilities and parameter definitions. We also describe the fitting procedure in detail.

Comment 4: If either p_walk or p_off is valid, does it mean they are either 0 or 1? Does the probability of transcription decrease?

Response: pwalk and poff are complementary probabilities, not binary switches. Their values are between 0 and 1 and sum to 1.

Comment 5: Figure 2 needs more explanation. Also, the note in C is not clear to me. It shows that gene 1 is being transcribed, but you wrote that gene 2 can only be transcribed when gene 1 isn’t transcribed.

Response: Thank you for pointing this out. We have revised the caption of Figure 3 to be more explicit (154-163).

Comment 6: Line 139: Please give more details on what the gradient data looks like (see comments in the method section).

Response: This is now described in more detail in the introduction section (lines 53-59).and data section (lines 297-305). There are also visualizations of these gradients in all 9 viruses in Figure 4, where both observed data and model predictions are presented for 9 NNSVs.

Comment 7: Figure 5A/B: What do those abbreviations mean (figure titles)?

The abbreviations are the order of the 5 genes in the VSV genome arranged from 3' end to 5' end. This is now clarified in the figure (lines 247-248).

Comment 8: Line 202: Explain the two data sets you use for model testing/validation. What were the two parameters you were using?

Response: We now explain the datasets in more detail (lines 228-230).

Comment 9: In the discussion section, you mention a model you briefly mentioned (ref 8). Please give more information about the model. It may be important to fit the data to that model and see whether the linear decrease in expression can explain the data. And from there, you could compare the linear with your exponential decrease model.

Response: We clarified that the cited work reported an empirical linear correlation between gene length and attenuation rather than a formal model fit. However, this also important to note that the attenuation is via a nonspecific mechanism, we would not expect a linear correlation, but rather an exponential relationship as we describe in our model. It may appear linear since most genes are roughly the same size with the L gene being a major outlier, but it should not be linear.

Comment 10: You also mention that degradation may impact protein levels. You should probably either include degradation in your model and the linear model (ref 8) or explain why you wouldn't include a degradation rate.

Response: We are only interested in modeling expression at the transcript level since we are interested in understanding the transcription mechanism itself. We thus do not include degradation as these transcripts have been observed to have the same degradation rate previously (lines 68-69). We use the protein level expression data for validation because there is not an equivalent dataset available for transcript level expression.

Reviewer #2:

Comment 1: It is assumed that the gene junctions are not different enough to explain the differences in transcription reinitiation and that "only RSV shows significant deviation specificity to the L-junction" (lines 77-78). However, there are decades-old articles deciphering the Gene End and Gene Start signals showing the relative importance of each nucleotide and that some residues are so important that their mutation can have a significant impact. In light of this, the last intergenic region of almost all viruses could be affected by such mutations. For instance, the last intergenic region of MeV is the only one for which the non-transcribed sequence is GCA and not the well-conserved GAA (by the way, the blue rectangle is not correctly placed on MeV in Fig. S1).

Response: We incorporated a more thorough and digested analysis of the previous literature into the introduction. We are not arguing that the gene junction do nothing and have no impact on expression, but rather that there is too much discourse in the literature (lines 82-92) to suggest that the variation within them is what truly underlies the gradient for all NNSVs. Furthermore, there is a lot of variation in these sequences even within the same virus (Fig. S1 and Fig 2). It seems unlikely that if they truly underlie such a consistent gradient in these viruses that these would be so diverse (lines 95-99).

Comment 2: There are studies on the intergenic regions that used bicistronic minigenome assays to analyze the reinitiation efficiency at the gene junction. For instance, in PMC5148173 (ref 47 in the manuscript), the gradient observed after switching the intergenic regions between the two reporters matches well the gradient observed in infected cells. The step at the gene junction is thus independent of the length of the genes. Such results, which contradict the model, are not discussed.

Response: Yes, they do observe attenuation effects from the different junctions, but they do not quite correspond to the expected attenuation levels. If they did we would see ~30% attenuation for all junctions except for the H-L junction which should have 80-90% attenuation. Instead, we see no attenuation (and even evidence of lower expression of the upstream gene than downstream gene) for junctions N-P and P-M, 50% attenuation for M-F and F-H junctions, and 60% attenuation for the H-L junction. Based on the provided distributions of these observations, these all would be a statistically significant deviation from our expectations. We extend the discussion of such phenomena (lines 82-92)

Comment 3: It is proposed that a transcribing polymerase has the same probability of falling off the template at any position on the mRNA. This would mean that the polymerase would make way more truncated mRNAs than full-length ones for long genes. For instance, for the L gene of VSV, it would mean that a bit less than 15 % of the polymerases that started transcription will make full-length mRNA (6380 nt-long). Thus, most of the polymerases (85 %) will generate truncated mRNAs. With such large amounts of non-polyadenylated mRNA, their lower stability seems insufficient to explain

why they would not be detectable. Moreover, such mRNA is not seen in in vitro transcription assays, which otherwise recapitulate well the current Start-Stop model.

Response: We acknowledge this discrepancy in our model. We believe that it is possible the lack of post-transcriptional modifications could result in a highly unstable RNA transcript that is degraded much faster than the mature transcripts (lines 262-265). Otherwise, there may be something present that neither the stop-start nor the nonspecific model are accounting for that explains this observation. We also suggest that the true mechanism may be a hybrid between these two models (lines 269-271).

Comment 3: Regarding the overlapping genes, the model proposing that the polymerases in the scanning mode (i.e., when in search for the next Gene Start signal after adding a polyA tail) can scan the genome in both directions and thus detect an upstream Start Signal (ref 18 in the manuscript) is never mentioned. Instead, this possibility is discarded without supporting this claim (lines 117-121).

Response: This is a valid criticism of the model. We make the assumption that movement of the polymerase occurs in a single direction. We now acknowledge this possibility and explain our choice to disregard it (lines 116-119).

Comment 5: The model proposed here does not take into account the efficiency of recognition of the Gene End sequence and assumes that all mRNA ends with a polyA (when full-length). However, there are numerous studies showing that the polymerase may fail to recognize the Gene End signal and generate readthrough mRNAs. These RNA products are not even mentioned.

Response: The purpose of the model is to provide a simplified explanation of the transcriptional gradient. As such, we intentionally omit some mechanisms and observations (lines 119-125). We discuss these in more detail now in the discussion as well as ways to incorporate them into the model (lines 272-274).

Comment 6: RSV does not belong to the Paramyxoviridae but Pneumoviridae family.

Response: We have fixed this classification error.

Reviewer #3:

Comment 1: Although the authors appreciate decades of studies addressing the NNSV transcription attenuation and they cite 20-30 papers related to that, in this reviewer’s opinion they failed in embracing all the nuances and molecular complexity that leads to successful transcription reinitiation of a downstream gene. For instance, the decisive role of successful 5′ cap formation by the L protein polymerase during each gene transcription initiation (Stillman and Whitt 1999, Ogino 2014, and others).

Response: We expand the discussion of all mechanisms associated with transcriptional that we do not explicitly model (lines 272-274). We are not saying they do not matter, but rather they may not be the defining feature that underlies the gradient (lines 119-125).

Comment 2: Given the kinetics of either a transcribing polymerase, or a polymerase paused in between the genes, quantified in Iverson and Rose 1981, it is unlikely that, as suggested by authors, polymerase exhibit comparable propensities to fall off at any given nucleotide position, both within genes and intergenic regions. Is that how the authors understand their poff parameter?

Response: We now more clearly present this finding in the introduction (lines 68-76) and acknowledge this as an open question in the discussion section (lines 263-265). The p_off parameter is the probability of the polymerase falling off the genome every step down the length of the genome.

Comment 3: It is also unclear where the values mentioned in lines 173-178 were derived from. Also, how the authors define and quantify the polymerase processivity?

Response: We now define processivity as the ability of the polymerase to stay associated with the viral genome (line 115).

Comment 4: Lines 137-138, it is unclear how the subtitle corresponds with the subsection below.

Response: We changed the name of this subsection to better reflect the content (line 162).

Comment 5: In line 195, the authors refer to Ball et al. 1999, regarding the effect of gene swapping in VSV. It seems that in this work though the P, M, and G genes were shuffled without changing the gene junction sequences. Authors should comment on other reports, for instance He and Lamb 1999 or Finke et al. 2000, where swapping the gene junction sequences indeed changed the levels of the downstream mRNA, irrespective of the gene position and the genome length.

Response: We now discuss these different findings in more detail in the introduction section (lines 82-92) and their importance in the discussion (lines 265-271).
