# Supplementary material for: The transcriptional gradient in negative-strand RNA viruses suggests a common RNA transcription mechanism
Source: PLoS Comput Biol. 2026 Jun 24;22(6):e1014441. doi: 10.1371/journal.pcbi.1014441 (PMC13313335; doi:10.1371/journal.pcbi.1014441)
Supplement: Attachment — Submitted filename: PLOS_Responses.pdf [file pcbi.1014441.s006.pdf]

Reviewer #1:

Comment 1: this reviewer is still concerned by some overinterpretations or misinterpretations of the literature. For instance, the authors state that "In RSV, similar assays yield conflicting results on whether intergenic regions drive attenuation (17, 20)". But this is misleading since these two studies did not compare the same thing. In ref 17, the authors did switch the intergenic region, i.e., the non-transcribed part of the gene junction, but in ref 20, the authors compared the whole gene junction, i.e., the gene end (GE) + intergenic region (IR) + gene start (GS). Therefore, ref 17 and ref 20 do not show "conflicting" results but rather complementary results.

This is correct. The manuscript has been updated to reflect this, highlighting these experiments as an example where the Stop-Start model works well (Lines 96-101).

Comment 2: Another example is the interpretation of the results of ref 24. The data in Figure 6b are normalized by the ratio found of the N-P junction. Hence, the fact that the ratio is higher for the P-M junction does not mean that there is a "higher downstream-than-upstream expression at the P-M junction" but rather that the attenuation is lower between P and M than between N and P. Also, the "only ~60% attenuation before L" is not absolute but corresponds to 60 % stronger attenuation than the one at the N-P junction.

This is correct and the claim has been rescinded.

Comment 3: In the next sentence, the authors state that "The presence of overlapping genes in RSV, EBOV, and MV further challenges the Stop-Start model, which assumes unidirectional transcription". Why would the Stop-Start model assume unidirectional transcription? In this model, once the polymerase has recognized a GE sequence, generated a polyA tail, and released the mRNA, it scans the genome to find another GS sequence. But why would the scanning be unidirectional? The review cited as a reference for the Start-Stop model (ref 19) clearly mentions that a polymerase can "scan backward to access a gs signal".

This is now updated in the manuscript. We no longer claim this challenges the Stop-Start model and our model now has a backtracking parameter instead of a parameter representing a probability of "missing" a transcriptional start signal (lines 169-172).

Comment 4: Finally, to complete the discussion, it might be worth mentioning that the "gradient" measured in infected cells is also affected by the intrinsic half-life of the individual mRNAs. Indeed, since in infected cells we only measure the accumulated mRNAs at a given time point and not the transcription activity itself, if a downstream mRNA is less expressed but more stable than its upstream one, it may seem expressed at the same level, or even at a higher level. Inversely, if an mRNA is well expressed but relatively unstable, the upstream gene junction will look like a strong attenuation step. Thus, the gradient measured by sequencing or radiolabeling of accumulated mRNAs is also affected by the differences in mRNA half-lives. This parameter may explain some of the discrepancies observed between minigenome systems and viruses. Indeed, in the bicistronic minigenome assays where the gene junctions are switched, the two mRNA stay mostly identical (except for a few mutations in the GS and GE), while in viruses, each mRNA is vastly different from the others. Hence, the differences in mRNA half-lives likely impact the mRNA ratios in infected cells much more than the ratios in minigenome assays.

We now suggest this in the discussion section (lines 317-320).

Reviewer #2:

Comment 1: Lines 39-40, 57-59 and throughout manuscript: Throughout the manuscript, the authors refer to the polymerase "falling off" or dissociating from the template. This is the terminology used in

earlier literature in the field and is a phenomenon that can occur with polymerases of positive strand RNA viruses. However, with recent breakthroughs in understanding of non-segmented negative strand RNA virus polymerase structure, it seems highly unlikely that the polymerase can dissociate from the template. Unlike the positive strand RNA virus polymerases, which have an open "right-hand" structure, the non-segmented negative strand RNA virus polymerases completely encircle the template, i.e., the template channel threads through the polymerase (e.g., Sala et al., 2025; PMID 40050611). The polymerase would need to undergo highly dramatic rearrangement, far greater than any structural variation that has been observed in the numerous polymerase structures available to date, to be able to dissociate from the template, except for unthreading when it reaches the template's 5' end. On the other hand, the polymerase could dissociate from the newly synthesized transcript, and there is ample evidence to indicate that this happens at the gene end signals, and during abortive RNA synthesis (e.g., in the event of capping failure). The available data indicates that polymerase that dissociates prematurely from the transcript cannot reinitiate at a gene start signal, likely because it needs to undergo the process of polyadenylation and transcript release to be able to recognize a gene start signal (likely because these processes cause necessary conformational changes in the polymerase). This aspect of polymerase structure and its relationship with the template doesn't necessarily change the conclusions of this manuscript, but the manuscript text should reflect the biological details of the transcriptional machinery.

This has now been added and the description of the model modified to better represent these findings (Lines 123-127, 140-144, as well as the model description section.

Comment 2: Lines 264-265 "Our model predicts abundant truncated transcripts, which are largely unobserved….it is possible that they ….are degraded much more rapidly than fully mature mRNAs". The original paper providing experimental evidence for the stop-start model (Iverson and Rose, 1981; PMID6258804) examined RNA made both in cells and in vitro (in which RNA degradation would be minimal). In addition, Iverson and Rose specifically looked for prematurely terminated RNA and could not detect it, whereas they could detect prematurely terminated RNA generated from a UV-exposed genome RNA. On the other hand, a study of RSV transcripts generated in vitro did identify prematurely terminated mRNAs (Liuzzi et al., 2005; PMID 16189012). It is not clear if this is due to differences in the approach or differences between the viruses. Regardless this point is not adequately addressed.

These results are now discussed more thoroughly in the article (lines 113-118, 311-318).

Comment 3: Measurement of mRNA transcript levels in a cellular environment is confounded by transcript stability. This is an issue with all experiments measuring steady state levels of different viral RNAs (e.g., RNA-seq data) and with any experiments in which gene junction substitutions affected the mRNA sequences. The situation in which intracellular mRNA stability is not a confounding factor is in experiments in which only the non-transcribed intergenic sequence was substituted and its effect on transcription examined. In this regard, reviewer 3 recommended that the authors examine a paper by Finke et al., 2000 which showed that changing the non-transcribed intergenic region in rabies virus impacted transcription of the downstream gene. This paper shows that for this virus, the intergenic region had a significant impact. The authors did not include rabies virus in their model fitting (Figure 4) and have not adequately addressed this key comment.

We agree that this provides a clear example of a system in which the Stop-Start model effectively explains transcriptional regulation. We have now incorporated this study into the revised manuscript (lines 96-98, 306-312), where we highlight it as a case in which intergenic sequence variation directly modulates transcription.

Regarding model fitting, we did not include rabies virus data in Figure 4 because our objective was not to exhaustively fit the model across all viral systems, but rather to test its applicability across representative datasets relevant to our study. While fitting the model to rabies virus transcription gradients is feasible, we instead discuss this system as an example where junction-specific effects dominate. To clarify this point, we explicitly acknowledge in the discussion that while the RAM model captures transcriptional behavior

across NNSVs, it does not universally account for all observed effects that could alter transcriptional gradients (lines 303-309).

Comment 4: The authors argue that the presence of overlapping genes is not consistent with the stop start model. Line 92-94: “The presence of overlapping genes in RSV, EBOV, and MV further challenges the stop-start model, which assumes unidirectional transcription”. This sentence conveys a lack of understanding of current models of mononegavirus polymerase behavior and the stop-start model of transcription. In considering the stop-start model it is important to distinguish between the polymerase in a transcribing mode in which the polymerase has the 3’ end of the transcript in its active site and is actively synthesizing RNA versus a scanning mode in which the polymerase does not have a transcript in its active site but remains associated with the template. In the transcribing mode, the polymerase can only move in a 3’ to 5’ direction relative to the template (it is “forced” to have a direction because of nucleotide addition onto the 3’ end of the transcript RNA), but in the scanning mode, there is nothing to determine in which direction the polymerase can move. The current model for how the polymerase accesses overlapping genes is that a transcribing polymerase releases the mRNA of the upstream gene at a gene end signal; having released the mRNA it becomes a scanning polymerase; it then can scan the template in both directions to find the next gene start signal and reinitiate. This model is supported by experimental evidence. E.g., examination of the mechanism by which the RSV L gene start signal is accessed showed that both the gene start and gene end signal of the upstream gene had to be intact for the polymerase to recognize the overlapping L gene start signal (Fearns and Collins, 1999; PMID 9847343). Similar results were also found in a study of Ebola virus overlapping gene junctions (Brauberger et al., 2015; PMID 26656691). The manuscript should be modified to acknowledge the experimentally derived data regarding gene overlaps.

This is now updated in the manuscript. We no longer claim this challenges the Stop-Start model and our model now has a backtracking parameter instead of a parameter representing a probability of “missing” a transcriptional start signal (lines 169-172).

Comment 5: Lines 82-99: In this paragraph, the authors point to apparent inconsistencies in the literature as justification for exploring an alternative model. However, this text contains numerous misconceptions and flaws. For example, this section cites Kuo et al., 1996 (PMID: 8709239) and Hardy et al.,1999 (PMID 9847319) as an argument that the literature on RSV is inconsistent. However, these papers examined different parts of the gene junction region. The manuscript by Kuo et al. examined the effect of substituting the intergenic region (i.e., the non-transcribed region that lies between the gene end and the gene start signals) whereas Hardy et al. examined the effect of switching out the gene junction (i.e., the gene end signal, the intergenic region, and the gene start signal). Kuo et al did not see an effect on the efficiency of upstream gene termination, whereas Hardy et al. did see an effect. The logical conclusion from comparing these papers is that in RSV the gene end signal is a key determinant of how efficiently the polymerase terminates mRNA synthesis at a gene junction, a conclusion which is borne out in another paper, also from Kuo et al. (Kuo et al., 1997, PMID: 9188557). Also note that the different groups used different experimental conditions that could have had additional effects. Hardy and coworkers were inadvertently using a mutant L for these studies that prevented efficient recognition of gene end signals – see Cartee et al., 2003; PMID 12805433 - and Kuo et al., were using virus superinfection to drive their minigenome, which meant that there was probably relatively limited M2-1 available; both these factors would affect levels of readthrough mRNA. Other evidence cited in this section also lacks attention to detail.

This section has been revisited and updated to more accurately reflect the literature (lines 96-101).

Comment 6: For example, the authors state “For EBOV, attenuation at the L junction is unremarkable, while the NP/VP35 and VP30/VP24 junctions paradoxically suppress upstream gene expression”. However, in this paper, the region that was substituted was not only the gene junction, but also the non-translated regions of the upstream and downstream genes. As these regions could have a significant impact on the stability of the mRNAs, it is difficult to draw conclusions regarding efficiency of

transcription across the gene junction from comparing the levels of the two mRNAs relative to each other (and this is not the point that the authors of the original paper were trying to make). Throughout the manuscript, the authors should pay more careful consideration of the prior literature, examining in detail exactly which sequences were substituted, which experimental system was used to interrogate them, and the fact that mRNA stability also needs to be considered. They should then integrate the findings regarding the effect of gene junction sequences into their model.

This is now mentioned in the manuscript (lines 153-156).

Comment 7: The authors acknowledge that co-transcriptional cap addition is a key process that could have a significant effect on mRNA synthesis. However, the significance of capping is not fully acknowledged in the manuscript. Multiple groups have shown that failure to cap causes abortive RNA synthesis. This could be a key factor causing attenuation at gene junctions.

The article now highlights this process more and even suggests that the observed nonspecific attenuation observed here may actually be mediated by this mechanism (lines 114-118, 311-312, 324-327)

Reviewer #3:

Comment 1: There are many examples, particularly in gene rearranged viruses where alterations to the L gene junction have affected its transcription levels. For example, doi: 10.1128/jvi.73.6.4705-4712.1999 and doi: 10.1128/JVI.73.8.6228-6234.1999. In the latter example, the MV H/L gene junction was altered by swapping out the upstream cistron resulting in a 4-fold difference in H/L gene expression ratios. Thus, L expression cannot be just a matter of how many nucleotides are being transcribed, This paper is mentioned, but not in any detail and it should be.

We now discuss the latter article in more detail in lines 106-110.

As for the first article, we are thoroughly reexamined it and were unable to find evidence that rearranging the genome impacted the relative expression of the L gene. Based on the figures quantifying protein expression in the article it appears the expression of the L gene does not change (Fig 4). There is also a plot where the authors show a gel with bands corresponding to the different mRNAs produced by the gene shuffled viruses, but they state that they were unable to quantify them due to a lack of resolution of the M and P mRNAs (Fig 3).

Comment 2: The model has a single value for P-off, but there are many examples where sequences are known to lead to altered attenuation. The two gene rearrangement papers, mentioned above for example, and there are many more. This should be stated, as surely it points to the new model being highly oversimplified.

This assumption is now explicitly discussed (lines 322-324).

Comment 3: I think the addition of a figure similar to the existing figure 4, showing how the stop/start model predicts mRNA levels and map onto the transcript data would be very useful.

Fitting the Stop-Start model would only yield perfect fits to the data because by definition, it essentially would suggest that the amount of attenuation at each junction would be the amount of attenuation that is observed. This is because it would have a single parameter for each junction.

We have now added a new portion of the manuscript where we fit both models to the Iverson and Rose data and then evaluate the models based on their predictive capabilities in the context of gene shuffled variants (Figure 6).

Comment 4: For figure 6, I am curious as to how the RNA quantities were acquired, as the levels are not stated in these papers.

We did not use RNA quantities since they were not quantified in this article. We used the protein levels reported in these articles. This is now discussed more explicitly (lines 253-257)

Comment 5: Line 95 – The statement, “Measles virus also deviates from the model, with higher downstream-than-upstream expression at the P-M junction and only ~60% attenuation before L” is not reflected in the corresponding figure 4.

This line originally was referring erroneously to the results of a different study (PMID: 27936158). This was a misinterpretation of the findings and the claim has been rescinded.
