# Supplementary material for: The transcriptional gradient in negative-strand RNA viruses suggests a common RNA transcription mechanism
Source: PLoS Comput Biol. 2026 Jun 24;22(6):e1014441. doi: 10.1371/journal.pcbi.1014441 (PMC13313335; doi:10.1371/journal.pcbi.1014441)
Supplement: Attachment — Submitted filename: PLOS_Response_to_Reviewers.pdf [file pcbi.1014441.s007.pdf]

Reviewer #2:

Comment 1. Line 138-129: "..formation of 5' cap during transcription is necessary for proper initiation and termination of transcription". Here, "initiation" should be "elongation".

This is now corrected (Line 117).

2. Line 252: "…overlapping occurs and the promoter of a gene (gene 2)…". Here "promoter" should be referred to as "transcription start site".

This is now corrected (Line 174).

3. Line 271: …"down the genome the termination signal (state 2)…". Here, there should be "to" inserted between "genome " and "the".

This is now corrected (Line 191).

4. Line 276 (Figure 3 legend): Here it should be stated that the two models are not mutually exclusive. It would be helpful if this was more clearly stated in the abstract and discussion sections too.

This has been updated to be more explicit in the respective areas of the article at (Lines 32-34, 198-199, 338-341).

5. Throughout the manuscript, virus names do not follow convention as set out by the ICTV (https://ictv.global/sites/default/files/web-files/General_Information/How-to-write-virus%2C-species%2C-and-other-taxa-names.2.pdf). For example, on lines 107-110, "Virus" should not be capitalized, and the only virus names in this list with capitalization should be Marburg and Ebola (these are place names, i.e., proper nouns whereas the other viruses in the list are not named based on proper nouns), on line 289, Pneumoviridae and other virus family names listed here should retain the capitalization but also be italicized (these are formal taxonomic names rather than virus names), line 434 "Rabies Virus" should be "rabies virus".

We appreciate this feedback and this is now fixed throughout the article.

6. As noted above, the two models proposed are not mutually exclusive and it is highly likely that both come into play during non-segmented negative strand RNA virus transcription. The discussion hints at this but it could be stated more clearly. To add weight to their argument, the authors might want to consider citing a review on the extensive work that has been done in other polymerase systems (e.g., Zhang and Landick, 2016, PMID: 26822487).

We thank the reviewer for this suggestion. The discussion now states this more clearly (Lines 338-341) and cite this article in the introduction (Lines 147-149).
