# Supplementary material for: The transcriptional gradient in negative-strand RNA viruses suggests a common RNA transcription mechanism
Source: PLoS Comput Biol. 2026 Jun 24;22(6):e1014441. doi: 10.1371/journal.pcbi.1014441 (PMC13313335; doi:10.1371/journal.pcbi.1014441)
Supplement: Peer review history

# Author comment: Author response to Decision Letter 0

**DOI:** 10.1371/journal.pcbi.1014441.r001

19 Sep 2025

Attachment Submitted filename: PLOS_Responses_v1.pdf

# Peer review: Decision Letter 0

**DOI:** 10.1371/journal.pcbi.1014441.r002

**Authors:** James R Faeder, Oleg A Igoshin

18 Nov 2025

PCOMPBIOL-D-25-01912

The Transcriptional Gradient in Negative-Strand RNA Viruses Suggests a Common RNA Transcription Mechanism

PLOS Computational Biology

Dear Dr. Peccoud,

Thank you for submitting your manuscript to PLOS Computational Biology. After careful consideration, we feel that it has merit but does not fully meet PLOS Computational Biology's publication criteria as it currently stands. Therefore, we invite you to submit a revised version of the manuscript that addresses the points raised during the review process.

Please submit your revised manuscript by Jan 18 2026 11:59PM. If you will need more time than this to complete your revisions, please reply to this message or contact the journal office at ploscompbiol@plos.org. When you're ready to submit your revision, log on to https://www.editorialmanager.com/pcompbiol/ and select the 'Submissions Needing Revision' folder to locate your manuscript file.

Please include the following items when submitting your revised manuscript:

* A rebuttal letter that responds to each point raised by the editor and reviewer(s). You should upload this letter as a separate file labeled 'Response to Reviewers'. This file does not need to include responses to formatting updates and technical items listed in the 'Journal Requirements' section below.

* A marked-up copy of your manuscript that highlights changes made to the original version. You should upload this as a separate file labeled 'Revised Manuscript with Track Changes'.

* An unmarked version of your revised paper without tracked changes. You should upload this as a separate file labeled 'Manuscript'.

If you would like to make changes to your financial disclosure, competing interests statement, or data availability statement, please make these updates within the submission form at the time of resubmission. Guidelines for resubmitting your figure files are available below the reviewer comments at the end of this letter

We look forward to receiving your revised manuscript.

Kind regards,

Oleg A Igoshin

Academic Editor

PLOS Computational Biology

James Faeder

Section Editor

PLOS Computational Biology

**Additional Editor Comments :**

In order to give the manuscript a proper consideration, we have decided to invite two reviewers who were not associated with the original decision but nevertheless top experts in the field. There are still concerns voiced about authors understanding or misinterpretations of the literature, taking the previously published data into consideration in developing thir model. However, in comparision to the previous version, the manuscript is significantly improved and we are happy to allow the authors to resubmit after major revision.

**Journal Requirements:**

1) Please ensure that the CRediT author contributions listed for every co-author are completed accurately and in full.

At this stage, the following Authors/Authors require contributions: Jean Peccoud. Please ensure that the full contributions of each author are acknowledged in the "Add/Edit/Remove Authors" section of our submission form.

The list of CRediT author contributions may be found here: https://journals.plos.org/ploscompbiol/s/authorship#loc-author-contributions

2) We ask that a manuscript source file is provided at Revision. Please upload your manuscript file as a .doc, .docx, .rtf or .tex. If you are providing a .tex file, please upload it under the item type u2018LaTeX Source Fileu2019 and leave your .pdf version as the item type u2018Manuscriptu2019.

3) Please upload all main figures as separate Figure files in .tif or .eps format. For more information about how to convert and format your figure files please see our guidelines:

https://journals.plos.org/ploscompbiol/s/figures

4) Some material included in your submission may be copyrighted. According to PLOSu2019s copyright policy, authors who use figures or other material (e.g., graphics, clipart, maps) from another author or copyright holder must demonstrate or obtain permission to publish this material under the Creative Commons Attribution 4.0 International (CC BY 4.0) License used by PLOS journals. Please closely review the details of PLOSu2019s copyright requirements here: PLOS Licenses and Copyright. If you need to request permissions from a copyright holder, you may use PLOS's Copyright Content Permission form.

Please respond directly to this email and provide any known details concerning your material's license terms and permissions required for reuse, even if you have not yet obtained copyright permissions or are unsure of your material's copyright compatibility. Once you have responded and addressed all other outstanding technical requirements, you may resubmit your manuscript within Editorial Manager.

Potential Copyright Issues:

i) Figure 3. Please confirm whether you drew the images / clip-art within the figure panels by hand. If you did not draw the images, please provide (a) a link to the source of the images or icons and their license / terms of use; or (b) written permission from the copyright holder to publish the images or icons under our CC BY 4.0 license. Alternatively, you may replace the images with open source alternatives. See these open source resources you may use to replace images / clip-art:

- https://commons.wikimedia.org

- https://openclipart.org/.

5) Please amend your detailed Financial Disclosure statement. This is published with the article. It must therefore be completed in full sentences and contain the exact wording you wish to be published.

1) State what role the funders took in the study. If the funders had no role in your study, please state: "The funders had no role in study design, data collection and analysis, decision to publish, or preparation of the manuscript."

6) Please revise your current Competing Interest statement to the standard "The authors have declared that no competing interests exist."

7) Please cite and label the supplementary tables in numerical order as “S1 Table” and “S2 Table,” and so forth.

Note: If the reviewer comments include a recommendation to cite specific previously published works, please review and evaluate these publications to determine whether they are relevant and should be cited. There is no requirement to cite these works unless the editor has indicated otherwise.

**Reviewers' comments:**

Reviewer's Responses to Questions

Reviewer #1: The authors took several of the reviewers’ comments into consideration and added enough caveats about their model to significantly improve the manuscript.

However, this reviewer is still concerned by some overinterpretations or misinterpretations of the literature. For instance, the authors state that “In RSV, similar assays yield conflicting results on whether intergenic regions drive attenuation (17, 20)”. But this is misleading since these two studies did not compare the same thing. In ref 17, the authors did switch the intergenic region, i.e., the non-transcribed part of the gene junction, but in ref 20, the authors compared the whole gene junction, i.e., the gene end (GE) + intergenic region (IR) + gene start (GS). Therefore, ref 17 and ref 20 do not show “conflicting” results but rather complementary results.

Another example is the interpretation of the results of ref 24. The data in Figure 6b are normalized by the ratio found of the N-P junction. Hence, the fact that the ratio is higher for the P-M junction does not mean that there is a “higher downstream-than-upstream expression at the P-M junction” but rather that the attenuation is lower between P and M than between N and P. Also, the “only ~60% attenuation before L” is not absolute but corresponds to 60 % stronger attenuation than the one at the N-P junction.

In the next sentence, the authors state that “The presence of overlapping genes in RSV, EBOV, and MV further challenges the Stop-Start model, which assumes unidirectional transcription”. Why would the Stop-Start model assume unidirectional transcription? In this model, once the polymerase has recognized a GE sequence, generated a polyA tail, and released the mRNA, it scans the genome to find another GS sequence. But why would the scanning be unidirectional? The review cited as a reference for the Start-Stop model (ref 19) clearly mentions that a polymerase can “scan backward to access a gs signal”.

Finally, to complete the discussion, it might be worth mentioning that the “gradient” measured in infected cells is also affected by the intrinsic half-life of the individual mRNAs. Indeed, since in infected cells we only measure the accumulated mRNAs at a given time point and not the transcription activity itself, if a downstream mRNA is less expressed but more stable than its upstream one, it may seem expressed at the same level, or even at a higher level. Inversely, if an mRNA is well expressed but relatively unstable, the upstream gene junction will look like a strong attenuation step. Thus, the gradient measured by sequencing or radiolabeling of accumulated mRNAs is also affected by the differences in mRNA half-lives. This parameter may explain some of the discrepancies observed between minigenome systems and viruses. Indeed, in the bicistronic minigenome assays where the gene junctions are switched, the two mRNA stay mostly identical (except for a few mutations in the GS and GE), while in viruses, each mRNA is vastly different from the others. Hence, the differences in mRNA half-lives likely impact the mRNA ratios in infected cells much more than the ratios in minigenome assays.

Reviewer #2: The non-segmented negative strand RNA viruses have a transcription gradient in which genes from the 3´ end of the genome are more abundant than those from the 5´ end. Early experiments by Iverson and Rose, 1981, using both VSV infected cells and an in vitro transcription assay showed that attenuation primarily occurred at the gene junctions (stop-start model). This early work has been largely supported in subsequent studies of the gene junction regions of non-segmented negative strand RNA viruses. In this resubmitted manuscript, King and coworkers present an alternative hypothesis, in which attenuation occurs at every nucleotide along the genome. They test this hypothesis by comparing their mathematical model with published data and it appears to fit.

It is entirely possible that there is some attenuation at each nucleotide and that the authors may be correct in their assertion that both their RAM model and the stop-start model models come into play. However, the authors minimize experimental data that demonstrates the key role that the gene junctions play and demonstrate misconceptions regarding published data. Without careful assessment of prior experimental data, and integration of these data into their model, it is difficult to assess how valid the RAM model is.

1. Lines 39-40, 57-59 and throughout manuscript: Throughout the manuscript, the authors refer to the polymerase “falling off” or dissociating from the template. This is the terminology used in earlier literature in the field and is a phenomenon that can occur with polymerases of positive strand RNA viruses. However, with recent breakthroughs in understanding of non-segmented negative strand RNA virus polymerase structure, it seems highly unlikely that the polymerase can dissociate from the template. Unlike the positive strand RNA virus polymerases, which have an open “right-hand” structure, the non-segmented negative strand RNA virus polymerases completely encircle the template, i.e., the template channel threads through the polymerase (e.g., Sala et al., 2025; PMID 40050611). The polymerase would need to undergo highly dramatic rearrangement, far greater than any structural variation that has been observed in the numerous polymerase structures available to date, to be able to dissociate from the template, except for unthreading when it reaches the template’s 5’ end. On the other hand, the polymerase could dissociate from the newly synthesized transcript, and there is ample evidence to indicate that this happens at the gene end signals, and during abortive RNA synthesis (e.g., in the event of capping failure). The available data indicates that polymerase that dissociates prematurely from the transcript cannot reinitiate at a gene start signal, likely because it needs to undergo the process of polyadenylation and transcript release to be able to recognize a gene start signal (likely because these processes cause necessary conformational changes in the polymerase). This aspect of polymerase structure and its relationship with the template doesn’t necessarily change the conclusions of this manuscript, but the manuscript text should reflect the biological details of the transcriptional machinery.

2. Lines 264-265 “Our model predicts abundant truncated transcripts, which are largely unobserved….it is possible that they ….are degraded much more rapidly than fully mature mRNAs”. The original paper providing experimental evidence for the stop-start model (Iverson and Rose, 1981; PMID6258804) examined RNA made both in cells and in vitro (in which RNA degradation would be minimal). In addition, Iverson and Rose specifically looked for prematurely terminated RNA and could not detect it, whereas they could detect prematurely terminated RNA generated from a UV-exposed genome RNA. On the other hand, a study of RSV transcripts generated in vitro did identify prematurely terminated mRNAs (Liuzzi et al., 2005; PMID 16189012). It is not clear if this is due to differences in the approach or differences between the viruses. Regardless this point is not adequately addressed.

3. Measurement of mRNA transcript levels in a cellular environment is confounded by transcript stability. This is an issue with all experiments measuring steady state levels of different viral RNAs (e.g., RNA-seq data) and with any experiments in which gene junction substitutions affected the mRNA sequences. The situation in which intracellular mRNA stability is not a confounding factor is in experiments in which only the non-transcribed intergenic sequence was substituted and its effect on transcription examined. In this regard, reviewer 3 recommended that the authors examine a paper by Finke et al., 2000 which showed that changing the non-transcribed intergenic region in rabies virus impacted transcription of the downstream gene. This paper shows that for this virus, the intergenic region had a significant impact. The authors did not include rabies virus in their model fitting (Figure 4) and have not adequately addressed this key comment.

4. The authors argue that the presence of overlapping genes is not consistent with the stop start model. Line 92-94: “The presence of overlapping genes in RSV, EBOV, and MV further challenges the stop-start model, which assumes unidirectional transcription”. This sentence conveys a lack of understanding of current models of mononegavirus polymerase behavior and the stop-start model of transcription. In considering the stop-start model it is important to distinguish between the polymerase in a transcribing mode in which the polymerase has the 3’ end of the transcript in its active site and is actively synthesizing RNA versus a scanning mode in which the polymerase does not have a transcript in its active site but remains associated with the template. In the transcribing mode, the polymerase can only move in a 3’ to 5’ direction relative to the template (it is “forced” to have a direction because of nucleotide addition onto the 3’ end of the transcript RNA), but in the scanning mode, there is nothing to determine in which direction the polymerase can move. The current model for how the polymerase accesses overlapping genes is that a transcribing polymerase releases the mRNA of the upstream gene at a gene end signal; having released the mRNA it becomes a scanning polymerase; it then can scan the template in both directions to find the next gene start signal and reinitiate. This model is supported by experimental evidence. E.g., examination of the mechanism by which the RSV L gene start signal is accessed showed that both the gene start and gene end signal of the upstream gene had to be intact for the polymerase to recognize the overlapping L gene start signal (Fearns and Collins, 1999; PMID 9847343). Similar results were also found in a study of Ebola virus overlapping gene junctions (Brauberger et al., 2015; PMID 26656691). The manuscript should be modified to acknowledge the experimentally derived data regarding gene overlaps.

5. Lines 82-99: In this paragraph, the authors point to apparent inconsistencies in the literature as justification for exploring an alternative model. However, this text contains numerous misconceptions and flaws. For example, this section cites Kuo et al., 1996 (PMID: 8709239) and Hardy et al.,1999 (PMID 9847319) as an argument that the literature on RSV is inconsistent. However, these papers examined different parts of the gene junction region. The manuscript by Kuo et al. examined the effect of substituting the intergenic region (i.e., the non-transcribed region that lies between the gene end and the gene start signals) whereas Hardy et al. examined the effect of switching out the gene junction (i.e., the gene end signal, the intergenic region, and the gene start signal). Kuo et al did not see an effect on the efficiency of upstream gene termination, whereas Hardy et al. did see an effect. The logical conclusion from comparing these papers is that in RSV the gene end signal is a key determinant of how efficiently the polymerase terminates mRNA synthesis at a gene junction, a conclusion which is borne out in another paper, also from Kuo et al. (Kuo et al., 1997, PMID: 9188557). Also note that the different groups used different experimental conditions that could have had additional effects. Hardy and coworkers were inadvertently using a mutant L for these studies that prevented efficient recognition of gene end signals – see Cartee et al., 2003; PMID 12805433 - and Kuo et al., were using virus superinfection to drive their minigenome, which meant that there was probably relatively limited M2-1 available; both these factors would affect levels of readthrough mRNA. Other evidence cited in this section also lacks attention to detail. For example, the authors state “For EBOV, attenuation at the L junction is unremarkable, while the NP/VP35 and VP30/VP24 junctions paradoxically suppress upstream gene expression”. However, in this paper, the region that was substituted was not only the gene junction, but also the non-translated regions of the upstream and downstream genes. As these regions could have a significant impact on the stability of the mRNAs, it is difficult to draw conclusions regarding efficiency of transcription across the gene junction from comparing the levels of the two mRNAs relative to each other (and this is not the point that the authors of the original paper were trying to make). Throughout the manuscript, the authors should pay more careful consideration of the prior literature, examining in detail exactly which sequences were substituted, which experimental system was used to interrogate them, and the fact that mRNA stability also needs to be considered. They should then integrate the findings regarding the effect of gene junction sequences into their model.

6. The authors acknowledge that co-transcriptional cap addition is a key process that could have a significant effect on mRNA synthesis. However, the significance of capping is not fully acknowledged in the manuscript. Multiple groups have shown that failure to cap causes abortive RNA synthesis. This could be a key factor causing attenuation at gene junctions.

Reviewer #3: I am a late comer to this review process of this manuscript, not being involved in the previous round of reviewing. This manuscript is interesting and provocative, and I can see from previous reviewer comments that there was consensus in that the conclusions of a previous version were a little overstated, with most of these claims being softened in a revised version.

In my opinion, the biggest fail of the previous stop/start model is in explaining transcription of the L gene, always in the promoter distal position of NSNS RNA viruses. To the best of my knowledge, transcription of this gene across all NSNS viruses is always lower than predicted by the stop/start model, inconsistent with the simple explanation of attenuation localized to the gene junction alone. The results shown in figure 4 are interesting as this newer model appears to account for this low L gene transcription quite well.

It is a failing of the field that no-one has really made any serious attempts to unpick this L gene expression discrepancy, even though it would not be such a tricky thing to do. However, we do have the results from gene rearranged viruses and for these viruses, I feel the new model has not done such a good job in explaining their transcription characteristics.

Major comments:

1. There are many examples, particularly in gene rearranged viruses where alterations to the L gene junction have affected its transcription levels. For example, doi: 10.1128/jvi.73.6.4705-4712.1999 and doi: 10.1128/JVI.73.8.6228-6234.1999. In the latter example, the MV H/L gene junction was altered by swapping out the upstream cistron resulting in a 4 fold difference in H/L gene expression ratios. Thus, L expression cannot be just a matter of how many nucleotides are being transcribed, This paper is mentioned, but not in any detail and it should be.

2. The model has a single value for P-off, but there are many examples where sequences are known to lead to altered attenuation. The two gene rearrangement papers, mentioned above for example, and there are many more. This should be stated, as surely it points to the new model being highly oversimplified.

3. I think the addition of a figure similar to the existing figure 4, showing how the stop/start model predicts mRNA levels and map onto the transcript data would be very useful.

4. For figure 6, I am curious as to how the RNA quantities were acquired, as the levels are not stated in these papers.

5. Line 95 – The statement, “Measles virus also deviates from the model, with higher downstream-than-upstream expression at the P-M junction and only ~60% attenuation before L” is not reflected in the corresponding figure 4

**********

**Have the authors made all data and (if applicable) computational code underlying the findings in their manuscript fully available?**

The PLOS Data policy requires authors to make all data and code underlying the findings described in their manuscript fully available without restriction, with rare exception (please refer to the Data Availability Statement in the manuscript PDF file). The data and code should be provided as part of the manuscript or its supporting information, or deposited to a public repository. For example, in addition to summary statistics, the data points behind means, medians and variance measures should be available. If there are restrictions on publicly sharing data or code —e.g. participant privacy or use of data from a third party—those must be specified.

Reviewer #1: None

Reviewer #2: Yes

Reviewer #3: Yes

**********

PLOS authors have the option to publish the peer review history of their article (what does this mean?). If published, this will include your full peer review and any attached files.

If you choose “no”, your identity will remain anonymous but your review may still be made public.

**Do you want your identity to be public for this peer review?**  For information about this choice, including consent withdrawal, please see our Privacy Policy.

Reviewer #1: No

Reviewer #2: No

Reviewer #3: No

[NOTE: If reviewer comments were submitted as an attachment file, they will be attached to this email and accessible via the submission site. Please log into your account, locate the manuscript record, and check for the action link "View Attachments". If this link does not appear, there are no attachment files.]

**Figure resubmission:**

While revising your submission, we strongly recommend that you use PLOS’s NAAS tool (https://ngplosjournals.pagemajik.ai/artanalysis) to test your figure files. NAAS can convert your figure files to the TIFF file type and meet basic requirements (such as print size, resolution), or provide you with a report on issues that do not meet our requirements and that NAAS cannot fix.

After uploading your figures to PLOS’s NAAS tool - https://ngplosjournals.pagemajik.ai/artanalysis, NAAS will process the files provided and display the results in the "Uploaded Files" section of the page as the processing is complete. If the uploaded figures meet our requirements (or NAAS is able to fix the files to meet our requirements), the figure will be marked as "fixed" above. If NAAS is unable to fix the files, a red "failed" label will appear above. When NAAS has confirmed that the figure files meet our requirements, please download the file via the download option, and include these NAAS processed figure files when submitting your revised manuscript.

**Reproducibility:**

To enhance the reproducibility of your results, we recommend that authors of applicable studies deposit laboratory protocols in protocols.io, where a protocol can be assigned its own identifier (DOI) such that it can be cited independently in the future. Additionally, PLOS ONE offers an option to publish peer-reviewed clinical study protocols. Read more information on sharing protocols at https://plos.org/protocols?utm_medium=editorial-email&utm_source=authorletters&utm_campaign=protocols

# Author comment: Author response to Decision Letter 1

**DOI:** 10.1371/journal.pcbi.1014441.r003

11 Apr 2026

Attachment Submitted filename: PLOS_Responses.pdf

# Peer review: Decision Letter 1

**DOI:** 10.1371/journal.pcbi.1014441.r004

**Authors:** James R Faeder, Eric C. Dykeman

5 Jun 2026

PCOMPBIOL-D-25-01912R1

The Transcriptional Gradient in Negative-Strand RNA Viruses Suggests a Common RNA Transcription Mechanism

PLOS Computational Biology

Dear Dr. Peccoud,

Thank you for submitting your manuscript to PLOS Computational Biology. After careful consideration, we feel that it has merit but does not fully meet PLOS Computational Biology's publication criteria as it currently stands. Therefore, we invite you to submit a revised version of the manuscript that addresses the points raised during the review process.

Please submit your revised manuscript by Aug 05 2026 11:59PM. If you will need more time than this to complete your revisions, please reply to this message or contact the journal office at ploscompbiol@plos.org. When you're ready to submit your revision, log on to https://www.editorialmanager.com/pcompbiol/ and select the 'Submissions Needing Revision' folder to locate your manuscript file.

Please include the following items when submitting your revised manuscript:

* A letter that responds to each point raised by the editor and reviewer(s). You should upload this letter as a separate file labeled 'Response to Reviewers'. This file does not need to include responses to formatting updates and technical items listed in the 'Journal Requirements' section below.

* A marked-up copy of your manuscript that highlights changes made to the original version. You should upload this as a separate file labeled 'Revised Manuscript with Track Changes'.

* An unmarked version of your revised paper without tracked changes. You should upload this as a separate file labeled 'Manuscript'.

If you would like to make changes to your financial disclosure, competing interests statement, or data availability statement, please make these updates within the submission form at the time of resubmission. Guidelines for resubmitting your figure files are available below the reviewer comments at the end of this letter.

As the corresponding author, your ORCID iD is verified in the submission system and will appear in the published article. PLOS supports the use of ORCID, and we encourage all coauthors to register for an ORCID iD and use it as well. Please encourage your coauthors to verify their ORCID iD within the submission system before final acceptance, as unverified ORCID iDs will not appear in the published article. Only the individual author can complete the verification step; PLOS staff cannot verify ORCID iDs on behalf of authors.

We look forward to receiving your revised manuscript.

Kind regards,

Eric C. Dykeman, Ph.D.

Academic Editor

PLOS Computational Biology

James Faeder

Section Editor

PLOS Computational Biology

**Additional Editor Comments:**

Dear authors

I realise that this manuscript has been under review for a while. My apologies for the delay.

However there are just minor typos / corrections that one of the reviewers pointed out that I believe are important to be addressed for accuracy of wording that is used in the virus field.

**Reviewers' comments:**

Reviewer's Responses to Questions

**Comments to the Authors:**

**Please note here if the review is uploaded as an attachment.**

Reviewer #1: I thank the authors for taking my comments into consideration.

Reviewer #2: This is a revised manuscript that provides a model to explain the transcription gradient that is characteristic of non-segmented negative strand RNA viruses. The authors have been responsive to prior critiques and have modified the manuscript to take into consideration details of previously published experimental data on this topic. The resulting manuscript is more nuanced and is a more accurate representation of the field and their findings. While the manuscript is largely sound overall, there are some minor editing errors that should be corrected.

Please note that line numbers refer to the marked-up version of the manuscript rather than the “clean” version.

1. Line 138-129: “..formation of 5’ cap during transcription is necessary for proper initiation and termination of transcription”. Here, “initiation” should be “elongation”.

2. Line 252: “…overlapping occurs and the promoter of a gene (gene 2)…”. Here “promoter” should be referred to as “transcription start site”.

3. Line 271: …”down the genome the termination signal (state 2)…”. Here, there should be “to” inserted between “genome “ and “the”.

4. Line 276 (Figure 3 legend): Here it should be stated that the two models are not mutually exclusive. It would be helpful if this was more clearly stated in the abstract and discussion sections too.

5. Throughout the manuscript, virus names do not follow convention as set out by the ICTV (https://ictv.global/sites/default/files/web-files/General_Information/How-to-write-virus%2C-species%2C-and-other-taxa-names.2.pdf). For example, on lines 107-110, “Virus” should not be capitalized, and the only virus names in this list with capitalization should be Marburg and Ebola (these are place names, i.e., proper nouns whereas the other viruses in the list are not named based on proper nouns), on line 289, Pneumoviridae and other virus family names listed here should retain the capitalization but also be italicized (these are formal taxonomic names rather than virus names), line 434 “Rabies Virus” should be “rabies virus”.

6. As noted above, the two models proposed are not mutually exclusive and it is highly likely that both come into play during non-segmented negative strand RNA virus transcription. The discussion hints at this but it could be stated more clearly. To add weight to their argument, the authors might want to consider citing a review on the extensive work that has been done in other polymerase systems (e.g., Zhang and Landick, 2016, PMID: 26822487).

Reviewer #4: King et al. present a model to describe the gradient in transcription of genes of nonsegmented negative-strand RNA viruses (NNSV). Their model uses one (or at most two) parameters to describe the process of attenuated expression, which has historically (since 1981) been described by four or more parameters based on sites of intergenic attenuation. The model is simple, elegant, and it performs well across multiple datasets of different NNSVs. Limitations of the model are clearly described.

**********

**Have the authors made all data and (if applicable) computational code underlying the findings in their manuscript fully available?**

The PLOS Data policy requires authors to make all data and code underlying the findings described in their manuscript fully available without restriction, with rare exception (please refer to the Data Availability Statement in the manuscript PDF file). The data and code should be provided as part of the manuscript or its supporting information, or deposited to a public repository. For example, in addition to summary statistics, the data points behind means, medians and variance measures should be available. If there are restrictions on publicly sharing data or code —e.g. participant privacy or use of data from a third party—those must be specified.

Reviewer #1: None

Reviewer #2: Yes

Reviewer #4: Yes

**********

PLOS authors have the option to publish the peer review history of their article (what does this mean?). If published, this will include your full peer review and any attached files.

If you choose “no”, your identity will remain anonymous but your review may still be made public.

**Do you want your identity to be public for this peer review?**  For information about this choice, including consent withdrawal, please see our Privacy Policy.

Reviewer #1: No

Reviewer #2: No

Reviewer #4: No

[NOTE: If reviewer comments were submitted as an attachment file, they will be attached to this email and accessible via the submission site. Please log into your account, locate the manuscript record, and check for the action link "View Attachments". If this link does not appear, there are no attachment files.]

**Figure resubmission:**

-->While revising your submission, we strongly recommend that you use PLOS’s NAAS tool (https://ngplosjournals.pagemajik.ai/artanalysis) to test your figure files. NAAS can convert your figure files to the TIFF file type and meet basic requirements (such as print size, resolution), or provide you with a report on issues that do not meet our requirements and that NAAS cannot fix.

After uploading your figures to PLOS’s NAAS tool - https://ngplosjournals.pagemajik.ai/artanalysis, NAAS will process the files provided and display the results in the "Uploaded Files" section of the page as the processing is complete. If the uploaded figures meet our requirements (or NAAS is able to fix the files to meet our requirements), the figure will be marked as "fixed" above. If NAAS is unable to fix the files, a red "failed" label will appear above. When NAAS has confirmed that the figure files meet our requirements, please download the file via the download option, and include these NAAS processed figure files when submitting your revised manuscript.

**Reproducibility:**

To enhance the reproducibility of your results, we recommend that authors of applicable studies deposit laboratory protocols in protocols.io, where a protocol can be assigned its own identifier (DOI) such that it can be cited independently in the future. Additionally, PLOS ONE offers an option to publish peer-reviewed clinical study protocols. Read more information on sharing protocols at https://plos.org/protocols?utm_medium=editorial-email&utm_source=authorletters&utm_campaign=protocols

# Author comment: Author response to Decision Letter 2

**DOI:** 10.1371/journal.pcbi.1014441.r005

11 Jun 2026

Attachment Submitted filename: PLOS_Response_to_Reviewers.pdf

# Peer review: Decision Letter 2

**DOI:** 10.1371/journal.pcbi.1014441.r006

**Authors:** James R Faeder, Eric C. Dykeman

12 Jun 2026

Dear King,

We are pleased to inform you that your manuscript 'The Transcriptional Gradient in Negative-Strand RNA Viruses Suggests a Common RNA Transcription Mechanism' has been provisionally accepted for publication in PLOS Computational Biology.

Before your manuscript can be formally accepted you will need to complete some formatting changes, which you will receive in a follow up email. A member of our team will be in touch with a set of requests.

Please note that your manuscript will not be scheduled for publication until you have made the required changes, so a swift response is appreciated.

IMPORTANT: The editorial review process is now complete. PLOS will only permit corrections to spelling, formatting or significant scientific errors from this point onwards. Requests for major changes, or any which affect the scientific understanding of your work, will cause delays to the publication date of your manuscript.

Should you, your institution's press office or the journal office choose to press release your paper, you will automatically be opted out of early publication. We ask that you notify us now if you or your institution is planning to press release the article. All press must be co-ordinated with PLOS.

Thank you again for supporting Open Access publishing; we are looking forward to publishing your work in PLOS Computational Biology.

Best regards,

Eric C. Dykeman, Ph.D.

Academic Editor

PLOS Computational Biology

James Faeder

Section Editor

PLOS Computational Biology

***********************************************************

Reviewer's Responses to Questions

**Comments to the Authors:**

**Please note here if the review is uploaded as an attachment.**

Reviewer #2: All the suggested revisions have been made.

**********

**Have the authors made all data and (if applicable) computational code underlying the findings in their manuscript fully available?**

The PLOS Data policy requires authors to make all data and code underlying the findings described in their manuscript fully available without restriction, with rare exception (please refer to the Data Availability Statement in the manuscript PDF file). The data and code should be provided as part of the manuscript or its supporting information, or deposited to a public repository. For example, in addition to summary statistics, the data points behind means, medians and variance measures should be available. If there are restrictions on publicly sharing data or code —e.g. participant privacy or use of data from a third party—those must be specified.

Reviewer #2: Yes

**********

PLOS authors have the option to publish the peer review history of their article (what does this mean?). If published, this will include your full peer review and any attached files.

If you choose “no”, your identity will remain anonymous but your review may still be made public.

**Do you want your identity to be public for this peer review?**  For information about this choice, including consent withdrawal, please see our Privacy Policy.

Reviewer #2: No

# Editor report: Acceptance letter

**DOI:** 10.1371/journal.pcbi.1014441.r007

**Authors:** James R Faeder, Eric C. Dykeman

PCOMPBIOL-D-25-01912R2

The Transcriptional Gradient in Negative-Strand RNA Viruses Suggests a Common RNA Transcription Mechanism

Dear Dr King,

I am pleased to inform you that your manuscript has been formally accepted for publication in PLOS Computational Biology. Your manuscript is now with our production department and you will be notified of the publication date in due course.

The corresponding author will soon be receiving a typeset proof for review, to ensure errors have not been introduced during production. Please review the PDF proof of your manuscript carefully, as this is the last chance to correct any errors. Please note that major changes, or those which affect the scientific understanding of the work, will likely cause delays to the publication date of your manuscript.

Soon after your final files are uploaded, unless you have opted out, the early version of your manuscript will be published online. The date of the early version will be your article's publication date. The final article will be published to the same URL, and all versions of the paper will be accessible to readers.

For Research, Software, and Methods articles, you will receive an invoice from PLOS for your publication fee after your manuscript has reached the completed accept phase. If you receive an email requesting payment before acceptance or for any other service, this may be a phishing scheme. Learn how to identify phishing emails and protect your accounts at https://explore.plos.org/phishing.

Thank you again for supporting PLOS Computational Biology and open-access publishing. We are looking forward to publishing your work!

With kind regards,

Janani Seenivasan

PLOS Computational Biology | Carlyle House, Carlyle Road, Cambridge CB4 3DN | United Kingdom ploscompbiol@plos.org | Phone +44 (0) 1223-442824 | ploscompbiol.org | @PLOSCompBiol
